# Improving Public Health Intervention Design for Food‐Borne Zoonotic Disease Control: Insights From a Situational Analysis of Meat Consumers’ Knowledge and Practices in Burkina Faso

**DOI:** 10.1002/puh2.70269

**Published:** 2026-05-05

**Authors:** Madi Savadogo, Malik Orou Seko, Inoussa Conombo, Guesrim Lallogo, Serge Diagbouga, Zékiba Tarnagda, Rianatou Bada Alambedji

**Affiliations:** ^1^ Unité de Recherche sur Les Maladies à potentiel épidémique, Maladies émergentes et Zoonoses, Département Biomédical et Santé Publique Institut de Recherche en Sciences de La Santé (IRSS/CNRST) Ouagadougou Burkina Faso; ^2^ Service de Microbiologie‐Immunologie et Pathologie Infectieuse, Département de Santé Publique Et Environnement Ecole Inter‐Etat des Sciences et Médecine Vétérinaires Dakar Senegal; ^3^ Direction de La Santé Animale, Direction Générale des Services Vétérinaires Ministère de l'Agriculture, Des Ressources Animales et Halieutiques Ouagadougou Burkina Faso; ^4^ Fundamental and Applied Research for Animals and Health, Faculty of Veterinary Medicine University of Liege Liege Belgium; ^5^ Service d'economie Rurale et Gestion Ecole Inter‐Etat des Sciences et Médecine Vétérinaires Dakar Senegal

**Keywords:** Burkina Faso, consumer practices, food safety, meat hygiene, public health, zoonoses

## Abstract

**Background:**

In low‐ and middle‐income countries, meat consumption is often associated with health risks due to limited awareness of zoonotic disease transmission. Burkina Faso is not exempt from these public health threats. This study aimed to assess knowledge and practices related to meat‐borne zoonotic risks among meat consumers in Burkina Faso.

**Methods:**

A cross‐sectional descriptive and analytical survey was conducted from August to November 2022, including 849 participants. Data were collected using a structured questionnaire administered through face‐to‐face interviews. Scores were attributed to assess levels of knowledge and practices regarding zoonotic risks associated with meat handling and consumption. Statistical analysis was performed using descriptive statistics and chi‐square and Fisher's exact tests.

**Results:**

According to findings, although 90.22% acknowledged that meat could transmit zoonotic diseases to humans, only 31.22% could name at least one zoonotic disease. Poor hygiene in meat sale outlets was reported by 50.77% of respondents. Good practices, such as proper meat storage, were inconsistently reported, as 66.90% reportedly stored meat exposed to open air, and 46.29% used the same utensils for meat preparation and meal service in households. Overall, level of education, professional occupation, religion and position held in the household were significantly associated with both knowledge and practices (*p* < 0.05), and participants having better knowledge were more likely to adopt safer practices (*p* < 0.05).

**Conclusion:**

Despite a general awareness of meat‐related health risks, including zoonotic diseases, knowledge gaps and unsafe practices remain prevalent among consumers. Reducing meat‐borne zoonotic diseases requires shifting from general awareness to actionable, behaviour‐oriented interventions. By combining community education, improved market hygiene and multi‐sectoral collaboration, public health authorities can substantially reduce preventable exposure to zoonotic pathogens while preserving the nutritional benefits of meat consumption.

## Graphical abstract

This study highlights that most consumers recognize that meat can transmit zoonotic diseases. However, substantial gaps persist in specific knowledge and in the consistent adoption of safe handling practices. By combining community education, improved market hygiene and One Health initiatives, policy‐makers can substantially reduce exposure to zoonotic pathogens while preserving the nutritional benefits of meat consumption.

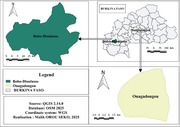

## 1 Introduction

Animal resources provide food products, such as eggs, milk and meat, which play an essential role in human nutrition by providing high‐quality proteins and essential micronutrients necessary for proper physiological functioning of the human body. Meat, in particular, is recognized as an important source of essential amino acids, bioavailable iron and zinc, B vitamins and other critical nutrients that contribute to growth, organ development, cognitive performance and overall health outcomes [1, 2].

Over recent decades, global meat demand has increased substantially, driven by demographic growth, urbanization, improved incomes among people, continuous lifestyle changes and evolving dietary preferences towards animal protein–rich food [3]. In response, livestock production systems have expanded and become diversified, whereas value chains have become increasingly complex. Indeed, meat and other products of animal origin pass through multiple stages—production, slaughter, processing, transportation, marketing and preparation—before reaching consumers. Although this transformation contributes to food availability and economic development, it also multiplies potential points of contamination along the value chains.

Meat is a highly sensitive to microorganisms and is particularly vulnerable to contamination by pathogens [4]. Contamination may arise from infected animals, lack of hygiene in slaughtering practices, inadequate storage conditions or improper handling and preparation of meat. Previous studies indicated that the health status of handlers, personal hygiene and food safety practices are key determinants in the transmission of food‐borne pathogens [5]. In the absence of adequate hygiene practices and sanitary controls, meat handling and consuming can therefore represent a significant pathway for the transmission of zoonotic diseases to humans.

In Burkina Faso, the national economy is mainly based on agro‐sylvo‐pastoral and fisheries activities and sustains the majority of the rural communities, which represent 72% of the national population [6]. The country exports livestock and products of animal origin to countries—such as Benin, Côte d'Ivoire, Ghana and Togo, as well as supplies the domestic demand for meat. Despite the economic and nutritional importance of livestock, many sanitary concerns persist at different stages of value chains. Unfortunately, a critical knowledge gaps remain. Indeed, whereas structural and regulatory aspects of meat hygiene and processing have received some attention in public policies, little is known about consumers’ knowledge, perceptions and everyday practices regarding zoonotic risks associated with meat handling and consumption. These gaps are particularly critical in the local context where informal markets and limited food safety oversight may increase exposure to food‐borne pathogens. Without a clear understanding of how consumers perceive and manage these risks, public health interventions and risk communication strategies may fail to address key social and behavioural drivers of food‐borne zoonotic pathogens. Although several studies have already addressed zoonotic disease control and prevention in the country [7, 8, 9], to the best of our knowledge, no study has specifically examined consumers’ knowledge and practices concerning zoonotic diseases transmitted through meat in the study sites. Therefore, addressing this gap has become essential for designing evidence‐based public health interventions aiming at reducing food‐borne zoonotic diseases. Therefore, this study aims to assess consumers’ knowledge and practices related to the risk of zoonotic disease transmission through the handling and consumption of meat in Burkina Faso.

## 2 Materials and Methods

### 2.1 Study Design and Location

**FIGURE 1:**
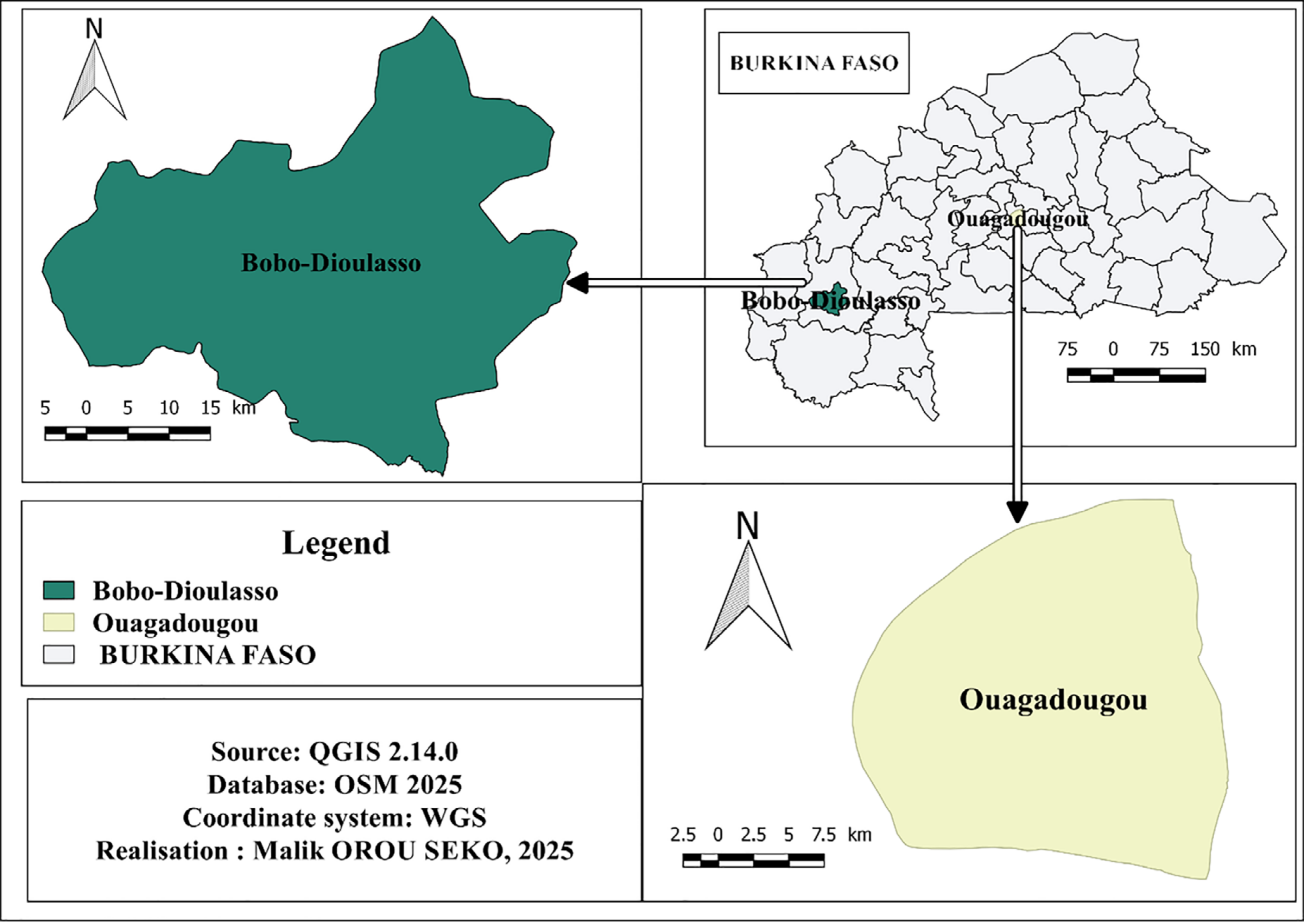
Geographical location of the two study areas.

A cross‐sectional descriptive and analytical survey was conducted from August to November 2022. It focused on the two main cities of Burkina Faso (Figure 1), namely, Ouagadougou (the administrative capital of the country) and Bobo Dioulasso (the economic capital of the country). The country covers an area of approximately 274,200 km^2^ with an estimated population of over 22 million inhabitants [6]. The two selected cities are the most densely populated and host the largest slaughterhouses of the country.

### 2.2 Study Population and Sampling Method

The study included households and meat trading outlets in the cities of Ouagadougou and Bobo Dioulasso. In the selected areas, surveys were conducted in households and meat trading outlets. Interviews targeted individuals aged 18 years and over. A simple random sampling method was used. Indeed, in each study site, a street was randomly selected, and a first household was randomly chosen, from which one household member was surveyed. From that point, every household or meat trading outlet located in the fourth position was included in the study. The sample size was estimated on the basis of the following formula:

$$n=\frac{{\left(Z_{\alpha /2}\right)}^{2}\times p\times \left(1-p\right)}{\varepsilon^{2}}$$

where *n* is the minimum required sample size; *p* is the estimated proportion of individuals perceiving a risk of contamination by pathogens related to meat consumption within the study population. In the absence of prior local data, we used 53.8%, based on the study conducted by Odetokun et al. in Nigeria [10]. This study assessed similar outcomes (consumers’ attitudes and risk perception of retail meat safety, it also evaluated the hygiene practices associated with retail meat sold to consumers within the Ilorin metropolis in Nigeria) in a comparable context. Indeed, Nigeria shares similar socio‐economic characteristics, food consumption patterns, informal meat distribution systems and public health challenges with Burkina Faso; *ε* = the margin of error was set at 0.05; ***Z*** α⁄2 = the standard normal value corresponding to a 95% confidence level was 1.96. Applying this formula resulted in a minimum sample size of 382 individuals to be surveyed. In total, 849 individuals were surveyed. The final sample of 849 participants therefore exceeded the minimum requirement of 382 individuals, ensuring adequate statistical precision and power. Moreover, this sample size increases the reliability of the estimated associations between explanatory variables and knowledge and practices.

### 2.3 Data Collection

A structured questionnaire was developed and administered to meat consumers both in selected households and meat trading outlets. The questionnaire comprised three main sections: The first section was related the socio‐economic and demographic characteristics of participants; the second section focused on knowledge of participants regarding food‐borne diseases and transmission patterns associated with meat handling and consumption; and the third section focused on practices related to the consumption of meat. Prior to the administration of the questionnaire, a pilot test was conducted among meat consumers, including 55 participants. Data were collected through face‐to‐face interviews conducted in the most three locally spoken languages (Mooré, Dioula and French) depending on the preference of each participant.

### 2.4 Data Processing and Statistical Analysis

The collected data were entered into Microsoft Excel 2016 spreadsheets for processing and recoding of variables. Data were then cleaned, processed and exported in R4.1.2 software for appropriate descriptive and analytical statistics. To assess the participants’ knowledge and practices regarding the risks of zoonotic diseases transmission associated with meat handling and consumption, a scoring model was developed and used to attribute weights according to the accuracy and completeness of answers as well as adequacy of practices reported. First, good practices related to behaviours and correct answers related to knowledge concerning zoonotic disease risks were defined by the research team. Then, answers from participants were analysed, and scores ranging from 0 to 3 were accordingly attributed (as summarized in Table 1). Subsequently, total scores were calculated for each participant regarding their knowledge and practices. On the basis of these total scores, participants were classified according to their level of knowledge and the adequacy of their practices [11, 12]. Finally, participants were categorized as follows [13]:

- Concerning the knowledge assessment, participants were grouped into three categories: unsatisfactory level of knowledge (if total score ranged from 0 to 1), satisfactory level of knowledge (if total score ranged from 2 to 4) and very satisfactory level of knowledge (if total score ranged from 5 to 7).
- Regarding practices, participants were grouped into two categories: poor practices (if total score ranged from 0 to 1) and good practices (if total score ranged from 2 to 3).

**TABLE 1:** Questions and scores used to assess knowledge and practices of participants (*n* = 849).

Variables	Example of answers	*N* observed	Corresponding scores
**1. Questions used to assess the participants’ level of knowledge**			
1.1. Do you think meat can be a source of infectious diseases in humans?			
Don't know/No	Don't know/No	83	0
Yes	Yes	766	1
1.2. Do you know examples of diseases that can be transmitted to humans through meat and other animal products?			
Incorrect answer	Don't know, gout disease, diabetes, haemorrhoid	584	0
One disease named	Tuberculosis, anthrax, cysticercosis, rabies, salmonellosis, high pathogen avian influenza, brucellosis	109	1
Two diseases named	Tuberculosis, anthrax, cysticercosis, rabies, salmonellosis, high pathogen avian influenza, brucellosis	32	2
Three diseases and more named	Tuberculosis, anthrax, cysticercosis, rabies, salmonellosis, high pathogen avian influenza, brucellosis	2	3
1.3. Under what circumstances can meat be a source of disease transmission to humans?			
Incorrect answer	Don't know	61	0
One correct answer	Consumption of contaminated meat, Handling contaminated animal products	627	1
Two correct answers	Consumption of contaminated meat, Handling contaminated animal products	81	2
1.4. What are the causes of poor meat quality?			
Incorrect answer	Don't know	371	0
Correct answer	Lack of training for traders, unhealthy state of the slaughtered animal, poor hygiene at slaughter and meat marketing outlet	478	1
**2. Questions used to assess the participants’ practices**			
2.1. How would you describe the way you use of utensils for meat preparation and meal service?			
Incorrect answer	Same utensils	393	0
Correct answer	Different utensils, same utensils after having cleaned them	456	1
2.2. How do you store meat before preparing or cooking it?			
Incorrect answer	Exposed to the open air	568	0
Correct answer	Drying, freezing, cooling	281	1
2.3. Under what storage conditions do you buy meat?			
Incorrect answer	Exposed on table	23	0
Correct answer	Stored in freeze, stored in fridge, displayed in a glass box	826	1

Descriptive statistics were used to estimate proportions, whereas analytical statistics (including the univariable Chi‐square test (*χ* ^2^) or the Fischer’ exact test) were performed to assess the association between explanatory variables (age, gender, education level, professional occupation, religion, marital status and position in household) and the level of knowledge as well as participants’ practices. The level of significance of the association was set at *p* < 0.05.

## 3 Results

### 3.1 Sociodemographic Characteristics of Participants

Sociodemographic characteristics are summarized in Table 2. Most of participants were female (64.07%). The study sample consisted mainly of young individuals, with 53.71% aged between 18 and 35 years. In terms of education level, 34.86% of participants had completed primary education, 28.03% had reached secondary level, and 9.19% had attained a university degree. However, a significant proportion (27.92%) of respondents had never attended school. Regarding professional status, 38.99% were housewives, whereas 42.40% worked in the private sector as employees. Concerning religion, Islam was the most represented faith (73.38%), followed by Christianity (25.91%).

**TABLE 2:** Sociodemographic characteristics of participants (*n* = 849).

Variables	Modalities	*N* observed	Percentages
Gender	Female	544	64.07
	Male	305	35.92
Age (in years)	18–35	456	53.71
	36–50	299	35.22
	51 and over	94	11.07
Education	Primary	296	34.86
	Secondary	238	28.03
	University	78	9.19
	No formal schooling	237	27.92
Professional occupation	Housewife	331	38.99
	Private sector employee	360	42.40
	Public sector employee	94	11.07
	Others^a^	64	7.54
Position in the household	Household head	259	30.51
	Spouse of the household head	426	50.18
	Others^b^	164	19.32
Religion	Christianism	220	25.91
	Muslim	623	73.38
	Traditionalism	6	0.71

^a^ Students, pupils.

^b^ Children, brothers, sisters or other relatives of the head of the household or of the spouse.

### 3.2 Knowledge About Risks Associated With Meat‐Borne Zoonotic Diseases

The majority of respondents were aware of the zoonotic risks associated with meat, with up to 90.22% stating that the handling and/or consumption of meat can cause disease in humans (Table 3). Regarding the circumstances of transmission, meat consumption was most frequently cited (81.53%), compared to handling (7.93%). When asked about diseases transmissible via contact with meat, 68.78% of respondents were unable to name at least one disease. Concerning hygiene in meat sale outlets, 50.77% of respondents considered it unsatisfactory, whereas only 5.89% judged it as very satisfactory. As for the perceived causes of poor meat quality, 29.56% of respondents attributed it to poor hygiene in slaughterhouses and sale outlets, and 15.67% to the unhealthy state of the slaughtered animals.

**TABLE 3:** Knowledge of participants about hygiene principles and zoonotic transmission risks associated with meat (*n* = 849).

Variables	Modalities	*N* observed	Percentages
Can diseases be transmitted from meat to humans?	No	48	5.65
	Yes	766	90.22
	Don't know	35	4.12
Under what circumstances can meat be a source of disease transmission to humans?	Consumption of contaminated meat	627	81.53
	Handling contaminated animal products	61	7.93
	Both	81	10.53
Do you know of any diseases that can be transmitted to humans from meat?	Incorrect answer	584	68.78
	One disease named	109	12.83
	Two diseases named	32	3.77
	Three diseases and more named	2	0.23
How would you describe the current state of hygiene in meat trading outlets?	Unsatisfactory	431	50.77
	Satisfactory	368	43.35
	Very satisfactory	50	5.89
What causes meat quality to be compromised?	Lack of training for traders	94	11.07
	Unhealthy state of the slaughtered animal	133	15.67
	Poor hygiene at slaughter and meat marketing outlet	251	29.56
	Don't know	371	43.70

### 3.3 Practices at Risk of Transmission of Meat‐Borne Zoonotic Diseases Among Participants

Beef was the most commonly consumed type of meat among surveyed households, accounting for 45.47% (Table 4). It was consumed more than twice as much as small ruminant meat (20.14%) and poultry (18.26%). Participants’ choices of meat type were influenced by several factors, including visual quality of meat (39.46%), availability of the meat near their households (37.34%). Regarding meat consumption frequency, 26.27% of households reportedly consumed meat occasionally, whereas 37.34% consumed it daily. The preferred sources of meat for households were meat retailing outlets (81.98%). Other reported sources included home or farm slaughtering (7.30%), purchasing directly from slaughterhouses (7.66%) and from grilled meat sale outlets locally known as dibiteries (3.06%). According to respondents, the criteria they used to assess hygiene and/or meat quality at points of sale included visual appearance (45.94%), presence of a veterinary stamp on carcasses (36.98%) and the use of packaging (17.08%). In their view, meat quality can be assessed through storage and presentation conditions (20.38%), personal hygiene of traders (36.28%) as well as transportation conditions (28.50%). Nevertheless, 88.57% expressed willingness to pay more if this guaranteed access to safer meat. In terms of meat storage before cooking, 66.90% of households reportedly left it exposed to air, 18.02% stored it in the refrigerator, and 9.78% used traditional storage techniques such as drying. As for cooking preferences, the majority (65.49%) favoured boiled meat, whereas 25.21% preferred grilled or braised meat, and 4.95% preferred fried meat. Regarding kitchen utensil use, 53.71% of respondents reported using separate utensils for meat preparation (cutting, washing) and for serving food. However, a notable proportion (46.29%) used the same utensils for these purposes.

**TABLE 4:** Practices at risk described among the participants (*n* = 849).

Variables	Modalities	*N* observed	Percentages
Preferred type of meat	Beef	386	45.47
	Small ruminant	171	20.14
	Pig	26	3.06
	Poultry	155	18.26
	Fish	111	13.07
Rationale for choice a type of meat	Availability in the locality Financial accessibility Perceived quality	317 197 335	37.34 23.20 39.46
Frequency of meat consumption	Every day	223	26.27
	Every 2 days	202	23.79
	Once a week	134	15.78
	Occasionally	290	34.16
Preferred mode of consumption	Boiled	556	65.49
	Fried	42	4.95
	Grilled or braised	214	25.21
	Others^a^	37	4.36
Place of meat purchase	Slaughterhouse	65	7.66
	Meat retailing outlet	696	81.98
	Dibitery	26	3.06
	Home or personal farm	62	7.30
Use of utensils for cutting meat and serving meals	Different utensils	456	53.71
	Same utensils after having cleaned them	393	46.29
Storage prior to preparation or cooking	Exposed to the open air	568	66.90
	Drying or fried	83	9.78
	Freezing	45	5.30
	Cooling	153	18.02
Criteria for judging meat hygiene in sale outlets	Normal visual appearance	390	45.94
	Presence of veterinary stamping	314	36.98
	Use of packaging	145	17.08
Criteria for assessing meat quality	Storage and presentation conditions at the sale outlet	173	20.38
	Personal hygiene and clothing for sales staff	308	36.28
	Meat hygiene and transport conditions	242	28.50
	State of health of slaughtered animal	126	14.84
Willingness to purchase good‐quality meat at a higher cost	Yes No	752 97	88.57 11.43

^a^ Dried meat, smoked meat and minced meat.

### 3.4 Factors Associated With Knowledge About Zoonotic Risks Related to Meat Handling and Consumption

Variables, such as level of education, professional occupation, position in household and religion of respondents, were significantly associated with level of knowledge regarding the risk of zoonotic disease transmission (*p* < 0.05, Table 5). However, sociodemographic characteristics, such as gender and age, were not significantly associated with the level of knowledge (*p* > 0.05).

**TABLE 5:** Association between explanatory variables and participants’ level of knowledge (*n* = 849).

Variables	Modalities	Level of knowledge described among participants			*p* value^a^
		Unsatisfactory	Satisfactory	Very satisfactory	
Gender	Female	21.79	40.63	1.65	0.09
	Male	13.78	20.49	1.65	
Age (in years)	18–35	18.49	33.21	2.00	0.88
	36–50	12.83	21.43	0.94	
	51 and over	4.24	6.47	0.35	
Education	Primary	16.25	18.49	0.11	0.00
	Secondary	9.06	17.31	1.65	
	University	1.77	6.24	1.17	
	No formal schooling	8.48	19.08	0.35	
Professional occupation	Housewife	15.42	23.20	0.35	0.00
	Private sector employee	14.61	27.21	0.58	
	Public sector employee	4.12	5.53	1.41	
	Others^b^	1.41	4.95	0.94	
Position in the household	Household head	12.25	16.96	1.29	0.00
	Spouse of the household head	18.14	31.21	0.82	
	Others^c^	5.18	12.96	1.17	
Religion	Christianism	12.37	12.48	1.06	0.00
	Muslim	23.20	48.06	2.12	
	Traditionalism	0.00	0.59	0.11	

^a^ Chi‐square test (*χ* ^2^) or Fischer’ exact test.

^b^ Students, pupils.

^c^ Children, brothers, sisters or other relatives of the head of the household or of the spouse.

### 3.5 Factors Associated With Risky Practices Related to Meat Handling and Consumption

Most sociodemographic characteristics, including age, level of education, religion, professional occupation and position in household, were significantly associated with meat handling and consumption practices (*p* < 0.05, Table 6). Gender was not significantly associated with practices (*p* > 0.05). Finally, the practices (good practices vs. poor practices) of the respondents were significantly associated with their level of knowledge regarding food‐borne zoonotic diseases (*p* < 0.05).

**TABLE 6:** Association between explanatory variables and practices among participants (*n* = 849).

Variables	Modalities	Practices described among participants		*p* value^a^
		Good practices	Poor practices	
Gender	Female	42.40	21.67	0.11
	Male	21.79	14.13	
Age (in years)	18–35	36.16	17.55	0.02
	36–50	20.49	14.72	
	51 and over	7.54	3.53	
Education	Primary	17.08	20.14	0.00
	Secondary	19.67	1.29	
	University	6.83	2.35	
	No formal schooling	20.61	7.30	
Professional occupation	Housewife	23.79	15.19	0.00
	Private sector employee	28.03	14.37	
	Public sector employee	6.24	4.83	
	Others^b^	6.12	1.41	
Position in the household	Household head	16.84	13.66	0.00
	Spouse of the household head	33.92	16.25	
	Others^c^	13.43	5.89	
Religion	Christianism	13.78	12.13	0.08
	Muslim	49.82	23.56	
	Traditionalism	0.59	0.12	
Level of knowledge	Unsatisfactory	16.37	19.19	0.00
	Satisfactory	44.64	16.49	
	Very satisfactory	3.18	0.12	

^a^ Chi‐square test (*χ* ^2^) or Fischer’ exact test.

^b^ Students, pupils.

^c^ Children, brothers, sisters or other relatives of the head of the household or of the spouse.

## 4 Discussion

This study aimed to assess consumers’ knowledge and practices regarding the risk of zoonotic disease transmission associated with the handling and consumption of meat in two cities of Burkina Faso. The results indicated that respondents strongly preferred beef, followed by meat from small ruminants, poultry and pork. These trends are consistent with findings from other studies, such as those conducted by Deneke et al. [14] and Oluwafemi et al. [15], which also reported a predominance of beef consumption. Globally, beef appears to be one of the most widely consumed meat, particularly due to its relative affordability for low‐income households and its broad availability. It is also culturally accepted in many regions of the world, as demonstrated by Oluwafemi et al. [16]. In terms of meat purchasing sites, most households reportedly bought their meat from meat retailing outlets. This finding is similar with reports from Ethiopia [14, 16, 17], which also found that meat retailing outlets were the most preferred meat purchasing sites. Indeed, in urban areas these outlets are widely distributed, making access easier for many households. Furthermore, consumers often develop trust‐based relationships with their meat retailers, which can come with advantages such as receiving a preferred piece or an extra piece of meat [18]. Regarding hygiene practices, most of respondents reported using separate utensils for meat preparation and meal serving. However, a significant proportion of households used the same utensils for both, which may indicate a lack of awareness regarding proper food handling practices or a limited access to adequate kitchen equipment. Indeed, the use of the same utensils for raw meat preparation and ready‐to‐eat food service reflects a potential risk of pathogen cross‐contamination of food within households [19, 20]. This result suggests either awareness gaps on indirect transmission pathways or structural constraints such as limited access to separate kitchen equipment. It also highlights the need for targeted food safety education focusing not only on general awareness on zoonotic disease prevention, but also on practical, low‐cost preventive measures adapted to household realities. As for meat preservation methods, it was reportedly exposed to air until consumption in the majority of households. Only 18.02% used cooling facilities, and 9.78% employed traditional technics such as meat drying. In northern Kenya, women have long used drying to preserve meat from microbiological spoilage [21, 22, 23]. However, whether reported traditional techniques are widely recognized and can be effective for short‐term preservation, they should be further evaluated and standardized for larger and safer use in households. Furthermore, the findings indicating that nearly 89% of respondents expressed a willingness to pay more for safer meat could represent a critical entry point for designing measures such as certification schemes or labelling strategies throughout the meat value chains. This dimension of willingness to pay more for safer meat need to be more investigated in the future studies. A study conducted in Senegal in outlets selling braised meat (commonly named dibiteries) also found similar results. Indeed, in this study, 84% of respondents were willing to pay an extra of $0.5–$0.84 on the purchase prices to improve the defects of the dibiterie meat quality [24].

Most of respondents acknowledged that meat handling and/or consumption can transmit diseases to humans. However, only 50.53% were able to name an example of a transmissible zoonotic disease. This gap reflects a limited understanding of zoonotic risks perception and concrete knowledge of transmission pathways despite a general awareness of potential threat. Such partial awareness may limit the effectiveness of preventive behaviours, as individuals may not fully understand the mechanisms through which contamination occurs. Targeted educational interventions should therefore aim to strengthen not only awareness but also specific disease‐related knowledge to support sustained behavioural changes. Similar trends were observed in Kenya and Tanzania [25, 26], though our observations remain higher than those reported in India, where only 16.4% were aware of zoonotic risks [27]. Zoonoses of bacterial (brucellosis, anthrax and tuberculosis), viral (rabies, high pathogen avian influenza) and parasite origin (cysticercosis) were cited most frequently. It is important to note that anthrax, rabies, brucellosis and high pathogen avian influenza are on the list of the five priority zoonotic diseases selected by decision‐makers in the framework of the national One Health multi‐sectoral commitment [28, 29]. The knowledge of zoonotic diseases as described in the study remain much lower than in Ethiopia, where taeniasis and anthrax were well known by community members [30, 31], likely due to higher levels of education in communities. Regarding meat hygiene in markets, 50.77% of participants considered it unsatisfactory. This result suggests substantial concerns regarding sanitary conditions at meat selling points. This consumers’ perception may reflect visible hygiene deficiencies such as inadequate storage, exposure to environmental contaminants and limited infrastructure. Although such awareness is encouraging, continued reliance on these markets likely reflects structural and economic constraints rather than informed choice. This highlights the need for systemic interventions targeting market infrastructure and regulatory enforcement in addition to consumer education. This is consistent with observations reported from Côte d'Ivoire [32], South Africa [33] and Benin [34], where poor hygiene was attributed to low education and lack of awareness among meat traders. Visual appearance and the presence of veterinary stamps on carcasses were the main hygiene criteria for consumers. Nevertheless, in a context characterized by an insufficient number of veterinary professionals to adequately cover the study area, some stamps may have been applied by unauthorized individuals. This is consistent with findings from South Africa and Senegal, where visual appearance heavily influences purchasing decisions among consumers [24, 35]. Although the use of packaging provides protection from contaminants and other types of biological and physical hazards, it was less mentioned. The limited mention of packaging as a protective measure suggests an under‐recognition of its role in preventing environmental risks and cross‐contamination of pathogens. Although cooking is often perceived as the primary safety measure, preventive practices during storage and marketing appear less adopted. This result underlined the need for food safety education interventions that emphasize simple, low‐cost protective measures adapted to local informal market and household realities.

Sociodemographic factors—especially level of education, religion, professional occupation, age and role in the household—were more likely to be associated with both level of knowledge and practices. Indeed, respondents with higher education levels demonstrated better knowledge as well as safer handling and consuming practices, corroborating findings from Ethiopia, Nigeria and Switzerland [36, 37, 38]. The observed association between education, occupation and safer practices could be directly translated into interventions implemented through schools, workplaces or targeted media campaigns aimed at informal sector workers. In contrast, sex and age were not consistently significant across studies as reported in previous studies [39].

Overall, although general awareness exists, practices are often guided more by subjective indicators like visual appearance than by verified safety standards, indicating the need of developing and communicating food safety awareness key messages in communities. Indeed, by improving public awareness, strengthening inspection capacities and promoting transparent communication through media are crucial steps towards safer meat handling and consumption.

Although the study provided relevant evidence, some limitations need to be mentioned. The most notable limitation concerns the partial application of the knowledge, attitudes and practices (KAP) model. Although the study provides valuable insights into knowledge and practices, it does not explicitly address attitudes, which are widely recognized as a crucial mediating factor shaping the translation of knowledge into practice. Attitudinal dimensions, such as perceptions of risk, willingness to modify behaviours or trust in public health and veterinary authorities, could either be directly integrated into the analytical framework in future investigations. Variables already included in the study, such as respondents’ willingness to pay more for higher quality meat or their perceptions of hygiene in markets, might be reinterpreted as proxies for attitudes. Similarly, the statistical analysis, while appropriately relying on descriptive statistics and bivariate tests, such as chi‐square and Fisher's exact tests, could be extended through the use of multivariable regression models. Such an approach would help disentangle the independent effects of sociodemographic characteristics on knowledge and practices and account for possible confounding variables. In addition, incorporating spatial analysis could strengthen future investigations, especially in exploring potential geographic disparities within the urban context. Future studies incorporating georeferenced data and spatial modelling technics would provide valuable complementary insights.

The inherent constraints of a cross‐sectional design, which preclude causal inference, the reliance on self‐reported practices that may be subject to desirability bias, the absence of direct observation of meat‐handling behaviours among consumers may also be limitations of this study.

Despite these limitations, the results already point to main actions for an improvement of food safety measures in the study areas. Three domains of action can be pinpointed as first steps:

1. **Mapping of stakeholders**: As part of efforts to strengthen the integration of food safety considerations into current national multi‐sectoral One Health initiatives, the funding could serve as a basis for identifying relevant stakeholders, clarifying their roles and responsibilities, and fostering coordinated actions to address health risks along the meat value chain in the country.
2. **Education and awareness raising**: This study pinpoints practical aspects to be included in communication and community engagement strategies: risks associated with meat being exposed to the open air, pathogens that can be transmitted to humans from products of animal origin, criteria or indicators of safe meat, and best practices for storing meat.
3. **Evidence and advocacy strengthening**: The sound functioning of meat inspection programmes implemented by veterinary services, generating strong evidences that describe the links, between hygiene standards, community awareness and the persistence of zoonotic risks associated with products of animal origin in the local context, would strengthen political commitment and stakeholder engagement. However, initial evidence has to be generated to initiate the virtuous cycle of food safety improvement. Interventional research programmes may play a key role in producing these policy‐relevant data. To combine this need with the previous point of awareness raising in the community, pilot studies could target community‐based surveillance of food‐borne zoonoses at different stages of the value chain: meat processing, meat transportation, meat marketing in outlets and meat preparation in households.

## 5 Conclusion

This study highlights that although most consumers recognize that meat can transmit zoonotic diseases, substantial gaps persist in specific knowledge and in the consistent adoption of safe handling practices. The limited ability to identify zoonotic diseases, the reported poor hygiene conditions in meat sale outlets, unsafe household storage practices and cross‐contamination risk during meal preparation collectively increase the risk of meat‐borne disease transmission. Importantly, the significant association between both knowledge and practices related to meat handling and consumption, and sociodemographic factors, such as the level of education, professional occupation and position in household, underscores the need for carefully designed interventions to strengthen food safety behaviours and mitigate public health risks.

Reducing meat‐borne zoonotic risks requires shifting from general awareness to actionable, behaviour‐oriented interventions. By combining community education, improved market hygiene and multi‐sectoral collaboration, public health authorities can substantially reduce preventable exposure to zoonotic pathogens while preserving the nutritional benefits of meat consumption.

## Author Contributions

**Zékiba Tarnagda**: methodology, writing – review and editing. **Malik Orou Seko**: software, formal analysis, methodology, writing – review and editing. **Serge Diagbouga**: writing – review and editing, methodology. **Madi Savadogo**: conceptualization, methodology, data curation, writing – original draft. **Rianatou Bada Alambedji**: conceptualization, methodology, validation, writing – review and editing, supervision. **Inoussa Conombo**: investigation, software, formal analysis, visualization, methodology, writing – review and editing. **Guesrim Lallogo**: investigation, methodology, visualization, software, formal analysis, writing – review and editing.

## Funding

The study was conducted through personal contribution from the authors. In addition, it was partially supported by Science for Africa Foundation to the Developing Excellence in Leadership, Training and Science in Africa (DELTAS Africa) programme [Afrique One‐ASPIRE, Del‐15‐008 and Afrique One‐REACH, Del‐22‐011].

## Ethics Statement

This study obtained ethics approval from the Research Ethical Committee of the Université Cheikh Anta Diop (Protocole‐0322/2018/CER/UCAD). Indeed, prior to administration of any questionnaire, participants were informed about the background and purpose of the study, highlighting that their participation was voluntary and all data will be kept anonymized as well as confidential. Therefore, only participants who agreed were interviewed.

## Conflicts of Interest

The authors declare no conflicts of interest.

## Data Availability

The data that support the findings of this study are available from the corresponding author upon reasonable request.
